# Enhanced Biodegradability in Soil of Chicken Feather by Steam Explosion for Potential Application in Agricultural Biodegradable Plastics

**DOI:** 10.3390/polym15183701

**Published:** 2023-09-08

**Authors:** Julen Vadillo, Sarah Montes, Hans-Jürgen Grande, Steven Verstichel, Jonna Almqvist, Krystyna Wrześniewska-Tosik

**Affiliations:** 1 CIDETEC, Basque Research and Technology Alliance (BRTA), Paseo Miramón, 196, 20014 Donostia-San Sebastian, Spain; 2 Advanced Polymers and Materials: Physics, Chemistry and Technology Department, University of the Basque Country (UPV/EHU), Avda. Tolosa 72, 20018 Donostia-San Sebastian, Spain; 3 Normec OWS, Pantserschipstraat 163, 9000 Gent, Belgium; 4 RISE Research Institutes of Sweden, Department of Biorefinery and Energy, S-892 50 Örnsköldsvik, Sweden; 5 Łukasiewicz Research Network, Łodz Institute of Technology, ul. Skłodowskiej-Curie 19/27, 90-570 Łódź, Poland

**Keywords:** chicken feather, biodegradation, steam explosion, disintegration, biopolymer

## Abstract

Feather waste is a major issue from an economic and environmental point of view. Even though there are already routes for the valorisation of feathers into fertilisers and feather meal, these are considered to have low added value. For more attractive applications, for example in agricultural biodegradable plastics, higher and faster degradability in soil is required. To face this challenge alternative approaches to accelerate biodegradation and disintegration processes are needed. In this context, steam explosion appears as an effective technology to modify the structure of feather and improve its soil degradability. In this work, chicken feathers were treated by steam explosion and the effect of treatment on their structure and physico-chemical and thermal properties were evaluated. Finally, the effect of the process conditions on the disintegration and biodegradation in soil of feathers was also investigated, finding an increased degradation in soil of steam explosion treated feathers. These results open up the possibilities of using feather waste as a component for environmentally friendly agricultural bioplastics that can be degraded in-situ in soil.

## 1. Introduction

The development of large-scale poultry farming, together with the increase in global poultry meat consumption (mainly chicken), has resulted in the generation of large quantities of feather waste. It is estimated that on a world scale, approximately an 8 × 10^5^ tonnes of chicken feather waste are produced per year [1], with their associated management costs. With some differences between countries, the current management practices of this waste include the disposal in landfills [2] or the burning of the waste [3], while a small portion is used in low added value applications such as animal feed or insulation materials [4]. Considering the current efforts by governments and organisations to promote circular bioeconomy and a zero-waste policy, it is of the utmost interest to find a way to manage feather waste in an economically and environmentally sustainable manner.

Chicken feathers (CF) are composed of over 90% protein, the main component being keratin, a fibrous and insoluble protein highly cross-linked mainly with disulphide [5,6]. Due to this strong bonding, their biodegradation in field is poor and slow. CF pose two different ordered conformations in their secondary structure; concretely, the polypeptide chain can be either folded into α-helix or bonded into plated sheets, which are denoted as β-sheets [7]. The latter is more abundant in hard tissues such as feathers, horns, or claws, whereas α-helix is usually found in soft tissues such as hair or wool [8]. Besides the ordered structures, a feather also includes some disordered structures, known as random coil, as well as chain reversal regions called β-turns between the β-sheets [9]. Feathers present a sulphur content that correspond mainly to covalent disulphide bonds. These bonds are formed between cysteines, which are present in the aforementioned α-helix and β-sheet structures and protect the feather against environmental degradation by heat, cold, light, water, or biological attack [10,11].

The traditional feather waste valorisation route is the conversion into feather meal to be utilised in animal feed [12]. For the generation of feather meal, CF have to be degraded by physical methods (pressurized hydrolysis and puffing) and chemical methods (acid and alkali) to make them digestible. However, these methods present some drawbacks such as high energy consumption during the production process and substantial damage to the obtained products [13]. Different authors have studied the effect of different processing parameters such as pH, temperature, addition of chemicals, and pressure on the nutritional value of feather meal [14,15]. According to their results, feather meal has the potential to be an important protein source for feedstock; however, it presents low digestibility, variable protein quality, and low nutrient bioavailability [16], which restricts its real applicability. An alternative valorisation route of CF widely studied is the extraction of keratin fibres due to the interesting properties they possess, such as low density, low toxicity, and abrasiveness, as well as high thermal insulation, flame resistance, and sustainability. Keratin fibres have been widely studied as reinforcement for the preparation of biocomposites [17,18]. 

Steam explosion (SE) process has been extensively used as a pre-treatment for wood, mainly to fractionate it into its three main components (cellulose, hemicellulose, and lignin) [19,20]. SE treatment of lignocellulosic biomass opens the fibres and makes the biomass components more accessible for subsequent processes [21]. The process consists of treating lignocellulosic biomass with hot steam (180 to 240 °C) under pressure (1 to 3.5 MPa) followed by an explosive decompression that results in a rupture of the rigid structure of fibres [22]. Apart for the thermal effect, as in traditional hydrothermal treatment, there is also a physical tearing effect that is accompanied by the rapid pressure release during the steam explosion process [23]. The SE process presents several advantages compared to other pre-treatment technologies for biomass, such as the use of water, avoiding other chemicals like acids, and the low corrosion of the equipment due to a mild pH of the reaction media when compared to acid hydrolysis processes [24]. Additionally, SE presents other benefits such as low capital investment, moderate energy requirements, and low environmental impact [25]. Considering the keratinous materials, the steam explosion of keratinous materials leads to disulphide bond cleavage and reduction of mechanical properties, moisture regain, and molecular weight, which affects the secondary structure of keratinous materials due to strong physical process parameters [26,27]. 

Thus, in this work, the effect of steam explosion treatment on chicken feathers has been investigated. For this purpose, CF were subjected to different SE processing conditions and characterized from the morphological, chemical, and thermal viewpoint. Additionally, the effect of SE treatment on the soil biodegradability of CF was also studied. It is expected that the steam explosion process will modify the structure of the feathers, modifying their disintegration capacity [28], and, thus, favouring its decomposition in natural soils.

## 2. Experimental

### 2.1. Obtention and Conditioning of Chicken Feathers

Raw chicken feathers were supplied by Cedrob (Ciechanów, Poland). Prior to the steam explosion, process feathers were conditioned by the following procedure. Initially, feathers were soaked and washed with detergent for 10 min (Dehaclin Fn 100 from CHT) and were posteriorly dried and stylized (40 min; T = 120 °C; P = 2 bar).

### 2.2. Steam Explosion and Processing of Chicken Feathers

The steam explosion process was carried out using a 40 L reactor designed for up to 28 bar and was heated with direct steam. The expansion vessel was 1000 L and was located below the rector to collect the material when the bottom valve in the reactor opened.

Feathers were introduced into the SE reactor and treated at different temperatures and residence times. In order to stablish a valid correlation between different combinations of conditions of SE process, a simple factor *R*_0_ was used [29]. This factor defines the severity of the SE process combining the influence of the two governing factors of the process, the temperature and the residence time, assuming a first order kinetics following the Arrhenius law as observed in the following equation:

(1) $$R_{0}=t\;e^{\frac{T-100}{14.75}}\;$$

where *T* is the SE temperature in °C, *t* is the residence time in minutes, and 14.75 the conventional energy of activation assuming a first-order reaction. The value of the severity factor is calculated by logarithmic operation.

(2) $$Severity=\log_{10}R_{0}$$

After the SE process, the obtained products were ground to obtain particles of 0.5 mm. Additionally, raw feathers were also ground at 0.5 mm for comparative purpose.

### 2.3. Characterization of Chicken Feathers

#### 2.3.1. Density of CF

The apparent density of CF was determined experimentally according to the ISO 9427 [30]. Five samples of each reference with a known volume were weighed and the density was determined as the ratio of the mass to volume. The average and standard deviation were reported. The statistical analysis of the results was performed using linear regression through the utilization of Origin 9.0 software.

#### 2.3.2. Fourier Transform Infrared Spectroscopy (FTIR)

The characteristic functional groups of chicken feathers treated at different SE conditions as well as of raw feathers were determined by Fourier transform infrared spectroscopy (FTIR) using a JASCO FT/IR-4100 (Easton, MD, USA) spectrometer in the range of 4000–400 cm^−1^ and 32 scans being the spectral resolution 1 cm^−1^.

The positions of the amide bands indicated the protein secondary structure [31]. Amide I is the combination of α-helix structure and β-sheet [32,33], whereas amide III can be assigned to α-helix, β-sheet structure as well as to β-turn and random coil [34]. The amide I region is most commonly used for secondary structure characterizations; however, due to overlapping peaks and possible interferences of water vibrational bands, the use of the amide III band is suggested for more accurate analysis of the protein secondary structure [35,36]. The secondary structures were assigned in amide III as α-helix (1330–1295 cm^−1^), β-turn (1295–1270 cm^−1^), random coil (1270–1250 cm^−1^), and β-sheet (1250–1220 cm^−1^) [35,37]. Hence, the deconvolution of the amide III band can give useful information about the structures presented in chicken feathers and their modification attributed to the SE process.

#### 2.3.3. Thermogravimetry (TGA)

The thermal stability was measured by thermogravimetric analysis using a TGAQ500 (TA Instruments, New Castle, DE, USA). Dynamic measurements were performed from 25 to 800 °C at a heating rate of 10 °C/min using constant nitrogen flow of 60 mL/min to prevent thermal oxidation processes of the polymer sample. The degradation temperature at a loss of 5% and 10% of the weight was determined as T_d5_ and T_d10_, respectively, which are typically studied parameters for studying the thermal stability of materials [38], whereas the maximum in the derivate weight of every degradation step was named as T_max_.

#### 2.3.4. Field Emission Scanning Electron Microscopy (FE-SEM)

CF morphology was analysed by field emission scanning electron microscopy. The microphotographs were taken with a Carl Zeiss Ultra Plus field-emission–scanning electron microscope (FE–SEM, Oberkochen, Germany) equipped with an energy dispersive X-ray spectrometer (EDXS). For the FE–SEM analysis, samples were previously coated with Au.

#### 2.3.5. X-ray Diffraction (XRD)

The effect of the SE process on the crystallinity of feathers was studied by X-Ray diffraction. The measurements were carried out using a Bruker D8 Discover diffractometer (Cu Kα radiation, λ = 0.154 nm, Billerica, MA, USA) equipped with a LynxEye PSD detector (Stockholm, Sweden). The diffractograms were recorded between 2θ = 5° and 80° at a scan speed of 0.003°/s.

The relative crystallinity of feathers was determined by deconvolution of the peaks of the diffractogram. Initially, the obtained difractograms were fitted to the Lorentz model by a curve fitting method, which is the most suitable model for this kind of material [39]. By calculating the area of the crystalline and amorphous profiles, the relative crystallinity of each sample was determined.

#### 2.3.6. Elemental Analysis

The amounts of carbon, nitrogen, hydrogen, and sulphur in raw feathers and feathers treated under different SE conditions were determined by elemental analysis. The measurements were carried out using an automatic elementary composition analyzer Vario Macro Cube of Elementar (Langenselbold, Germany).

#### 2.3.7. Biodegradation in Soil

The standard soil biodegradation test of the pol was performed according to ISO 17556 [40] but in duplicate instead of triplicate. This standard is used for testing the biodegradation of biodegradable materials [41]. In the test, the reference item cellulose and the test items were added as powder, directly mixed with standard soil and incubated in the dark at an ambient room temperature (25 °C ± 2 °C). Biodegradation is taking place through microbial activity and as a result, carbon dioxide and water is produced. The CO_2_ is captured in KOH and the CO_2_ production is regularly determined by titration, which allows calculating the cumulative CO_2_ production. The percentage of biodegradation can be calculated as the percentage of solid carbon of the test item, which has been converted to gaseous, mineral C under the form of CO_2_. In Figure S1, a diagram of the biodegradation test setup is displayed. A test item has demonstrated a satisfactory level of biodegradation when 90% absolute or relative biodegradation is reached. The maximum allowed test duration determined by this standard is two years.

## 3. Results and Discussion

### 3.1. Effect of SE on CF Density and Yield

The yield of the process in each case was calculated by mass difference between the raw feathers before SE treatment, and the obtained one after it. In Table 1, the prepared samples are displayed, detailing the steam explosion conditions, the obtained size after grinding as well as the final yield of every process.

**Figure 1:**
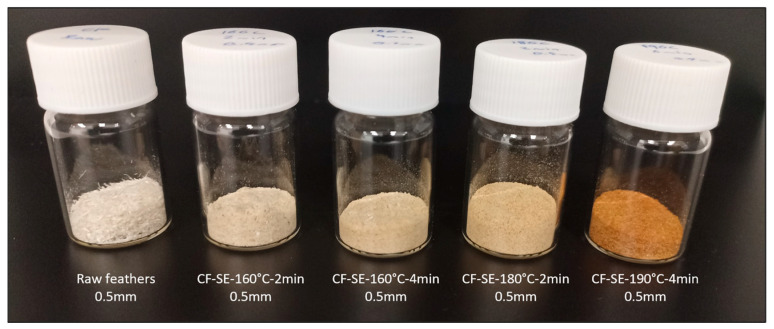
Digital images of the CF treated at different SE conditions.

The obtained product after the SE treatment presented a brownish colour, which became more intense as the severity factor of the process increased (Figure 1). This phenomenon was also observed in wood treated by SE [42]. In addition to this, a decrease of the yield as the severity factor of the SE process was observed, which has been previously observed for other materials [25,43]. This decrease of the yield seems to be more pronounced for severity factors above 3.

Regarding the bulk density, the results showed an increase of the apparent density as the severity factor of the SE process did. This result is in accordance with the ones observed for other types of natural materials such as lignocellulosic biomass [28,44], in which the increase of the severity of the conditions of the SE process resulted in a densification of the obtained material. In this case, the increase of the density can be fitted to a linear equation in the studied range obtaining a R^2^ of 0.95.

### 3.2. Chemical Structure of SE Feathers

**Figure 2:**
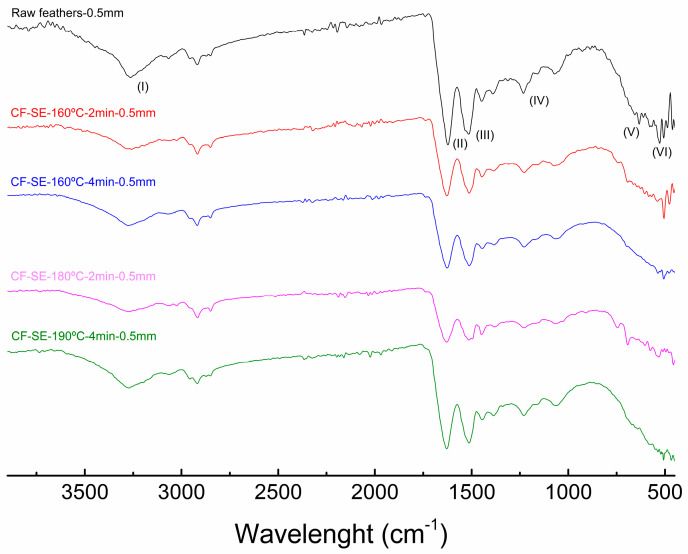
FTIR spectra of untreated chicken feather and feathers treated under different SE conditions.

The effect of the SE process on the chemical structure of chicken feathers was studied by FTIR. The spectra of the raw feather displayed in Figure 2 show the characteristic bands of the untreated CF: (I) hydrogen bonded N–H stretching vibration around 3300 cm^−1^ (Amide A band) [36], (II) C=O stretching and a minor contribution of N–H bending and C–N stretching between 1700–1600 cm^−1^ (Amide I band) [45,46], (III) N–H bending at 1540 cm^−1^ (Amide II band) [47], (IV) C–N stretching at 1240 cm^−1^ (Amide III band), and (V) N–H out-of-plane bending around 750–600 cm^−1^ [48]. Additionally, the band situated around 2900 cm^−1^ is related to symmetrical CH_3_ stretching vibration [47], whereas the small peak situated at 580 cm^−1^ is associated with S–S bonds [49].

Comparing the different obtained spectra, scarce modifications were observed as a result of the SE process. A slight decrease of the intensity of the peak assigned to S–S bonds (VI) is observed at 530 cm^−1^ in samples treated by steam explosion compared with the untreated ones. This reflects the disruption of the sulphur bonds as a result of the SE process [27].

Regarding the study of the different ratio of secondary structures present in the studied feathers, the peak analysis displayed in Table 2 revealed a higher amount of β-keratin compared with α-keratin, which is common in the case of feathers [50,51]. Additionally, some ordered secondary structures turned into unfolded or disordered ones after the SE process, as observed previously by other authors [52]. Concretely, a decrease of both β-sheet and β-turns structures were observed in systems treated by SE, leading to the increase of disordered random coil structures. This decrease of both β-sheet and β-turns is clear comparing the raw feathers with the ones treated with SE and seem to be higher with a more severe process. The increase of random coil, however, did not seem to increase clearly as the severity of the process did.

In contrast, also an increase of α-helix structure was observed in samples treated by SE. The β-sheet structures usually contain high amounts of cysteine that can interact, resulting in disulphide bonds, which have a negative effect on biodegradation [53]. The steam explosion process seems to disrupt the intermolecular bonding of the β-sheet leading to the apparition of higher amounts of random coil. This transition from β-sheet structures to random coil was also observed in other works as a result of a thermal treatment of the feathers [54], including the SE treatment [55]. Regarding the increase of the α-helix content, it is consistent with previous works, obtaining higher values of relative content as a result of the exposure at high temperatures [56], and concretely for a SE process [55].

### 3.3. Elemental Analysis of SE Feathers

Results for CHNS analysis of raw and treated feathers are shown in Table S1. In general, all samples presented an average composition of 47% C, 7.2% H, 15% N, and 2% S with the remaining 27% composed of oxygen and inorganic matter, which is typical for chicken feathers [57]. This nitrogen content suggests that chicken feathers can be used to produce bio-compost or animal feed, whereas the high carbon content may result in an easily available source of carbon for biodegradation agents such as micromycetes [58]. Additionally, the observation of sulphur suggests the presence of cysteine proteins, which can promote disulphide bonds between them. Comparing the effect of the SE process in the elemental composition of the feathers, no considerable differences were observed as far as nitrogen, carbon, and hydrogen is concerned; however, a decrease of the sulphur content is produced as the severity of the SE process increases. This can be attributed to the cleaving of the cysteine and the reaction of thiol groups, which are likely to further react, for example, to sulfoxyl compounds or formed sulphur containing volatiles [59]. Taking the aforementioned into account, it can be assumed that the disulphide crosslinking between cysteine is at least partly destroyed [55], which has an effect on the protein folding and on the association with the other polypeptide chains [60]. This decrease of the disulphide bond is more noticeable in feathers treated at more severe conditions such as in the case of CF-SE–190 °C–4 min–0.5 mm, and has a positive effect in the biodegradability of the material, as was observed previously [59].

### 3.4. Thermal Stability of SE Feathers

**Figure 3:**
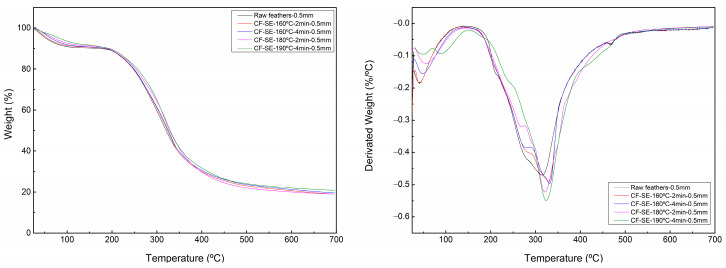
Degradation curves of feathers treated with different SE conditions (**left**) and derivative curves (**right**).

Thermogravimetry was used to evaluate the influence of SE treatment on the thermal stability of CF. Figure 3 shows the obtained degradation curves and their derivative in which a two-step degradation process was observed for all samples. The first step is attributed to the evaporation of moisture from feathers. This step is formed by three different types of water within chicken feathers, namely, free water, loosely bonded water, and chemically bonded water, which contribute to the conformational stability of keratin protein [61]. The second degradation step is aligned with the denaturation of the predominant β-sheet structure, skeletal degradation, and destruction of peptide bridge chain linkage, both hydrogen and disulphide bonds [61]. This region includes several chemical reactions and skeletal degradation by which keratins are decomposed to lighter products and volatile compounds such as H_2_S, CO_2_, H_2_O, and HCN [61].

Comparing the obtained results (Table S2), the aforementioned degradation steps presented a maximum in their derivative curve at higher temperatures, including the T_d5_ and T_d10_ as the severity of the SE process increased. This behaviour was observed previously in the lignocellulosic samples treated by SE, such as sugarcane [62] or broccoli wastes [63] and is attributed to the removal of low molecular weight compounds by the SE process. The differences between the treated and untreated feathers at Td_10_ is higher than the ones between the SE treated feathers at different severities. Additionally, the increase of the relative amount of α-helix, increase the thermal stability of the material, due to the greater packing efficiency of this secondary structure [61].

### 3.5. Morphology of SE Feathers

**Figure 4:**
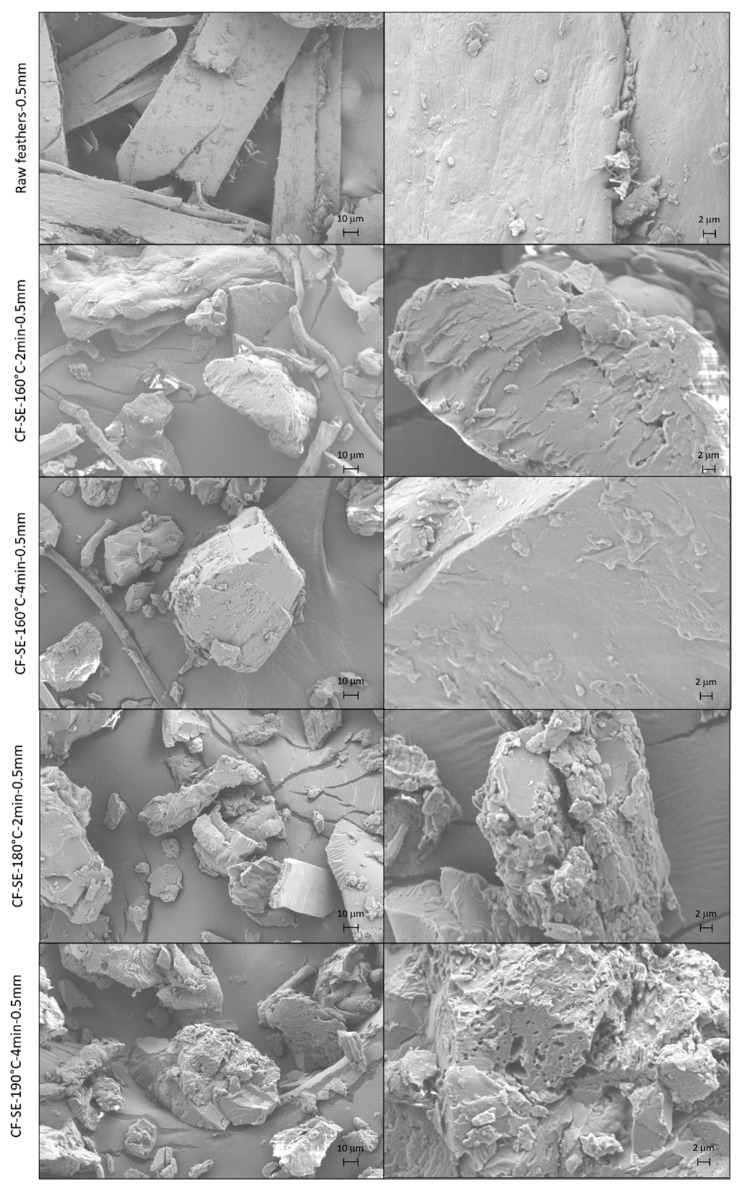
Images obtained by Scanning electron microscopy of the feathers treated at different steam explosion conditions. Magnification ×500 μm (**left**) and ×2000 μm (**right**).

In order to determine the effect of the SE on the morphology of the feathers, field emission scanning electron microscopy was used to obtain images of the different studied samples. Figure 4 shows the FE-SEM micrographs of treated feathers under different SE conditions, as well as raw feathers as control, showing in all cases a wide range of particle sizes. Additionally, as can be observed in the displayed images, an increase of the roughness is produced as the severity factor of the SE increase. This modification of the surface of the particles as a result of the SE treatment was reported previously for different materials [63,64], and is produced as a result of the physical process that took place during the tearing of the material and the posterior pressure release of the SE. This increase of the roughness is more noticeable in feathers treated under the most severe conditions (severity factor above 2.66) whereas in less treated feathers, the surface of the particles is still smooth. The increase of the roughness of the particle will increase the adhesion of microorganism to the feather, increasing biofouling [65] and, hence, favouring biodegradation.

### 3.6. X-ray Diffraction of the SE Feathers

**Figure 5:**
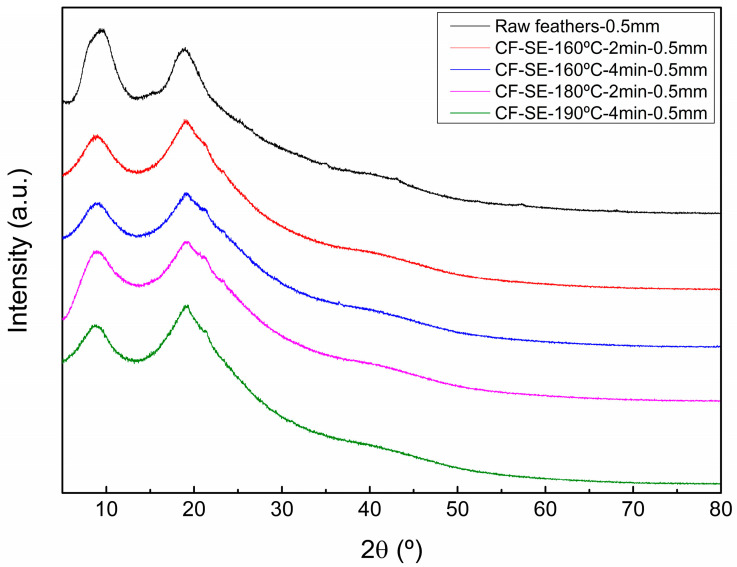
XRD diffractograms of raw and SE treated feathers.

Figure 5 shows the X-ray diffraction patterns of untreated chicken feathers and feathers treated by SE. The obtained diffractograms showed characteristic wide peaks around 9° and 19°. The former can be assigned to α-helix structure, whereas the latter is related to β-sheet structure [66,67]. Comparing the curves obtained for the different materials, nor apparition of new peaks nor displacement of the existing ones were observed, and all treated samples presented similar diffractograms.

The relative crystallinity of each sample was determined by the deconvolution of the diffractograms, and the obtained values are displayed in Table 3, and a representative deconvolution of each sample is displayed in Figure S2. The results showed an increase of the relative crystallinity for feathers treated under low SE conditions compared with the untreated ones, whereas in the case of samples subjected to SE presenting higher severity factors, a decrease of the crystallinity was observed. Less severe SE conditions led to the dissolution of amorphous zones and, thus, to the increase of the relative crystallinity, as observed by other authors for different types of lignocellulosic materials [68,69]. In more severe SE processes, however, as a result of the thermal degradation of the material, the relative crystallinity of the samples clearly decreased [63,70].

### 3.7. Biodegradation in Soil of SE Feathers

**Figure 6:**
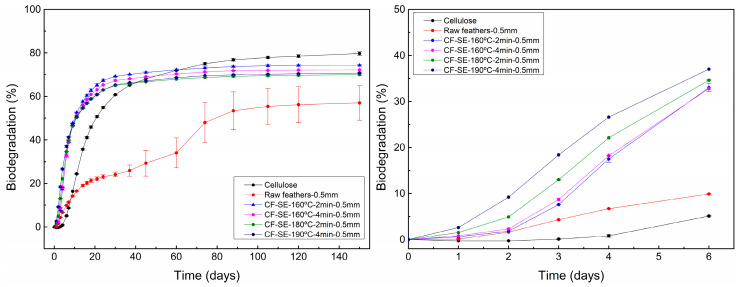
Biodegradation in soil curves of feathers with different SE conditions. Results up to 150 days (**left**) and six days (**right**).

Finally, the treated feathers in form of powder were mixed with standard soil and their biodegradation over time was measured to evaluate the effect of the SE treatment. The resulting curves, which are displayed in Figure 6 (left), showed a clear modification of the biodegradation behaviour of feathers as a result of the SE treatment. The untreated feathers presented a slow and gradual biodegradation with a maximum value of around 70% relative to the reference material (cellulose). The slow biodegradation reached a plateau around 120 days after the start of the test. In the case of feathers treated by SE, however, the biodegradation process took place rapidly, presenting a biodegradation rate higher than the raw feathers and even the reference material in the first 30 days. In all SE treated feathers, the plateau is reached after 30 days. After 150 days, relative biodegradation levels near 90% were measured, confirming that they were successfully degraded. This increase of both final biodegradation percentage and biodegradation rate can be explained by the transition from ordered β-sheet structures to disordered random coil ones, as was observed previously by FTIR as well as by the decrease of the disulphide bonds. The β-sheet structure has a significant effect on biodegradation, and its decrease leads to a faster and higher biodegradation [53], whereas the breaking of the disulphide bonds will also favour the biodegradation process [10].

Regarding the effect of the SE severity on the biodegradation of treated feathers, all studied samples presented similar biodegradation percentages at the plateau. According to the obtained results, the increase of the severity factor in the SE treatment seems to decrease the percentage of ordered structure as well as the sulphur linkages [59]. However, this modification of the inner structure of the feathers as the severity factor increased is not correlated with an increase of the total biodegradation of the material. Indeed, the less treated sample, that is, SE-160 °C–2 min–0.5 mm (SF = 2.07), showed the higher biodegradation percentage. Nonetheless, differences can be observed in the first steps of the biodegradation curves, concretely in the first five days. As can be observed in Figure 6 (right), feathers treated under more severe SE conditions presented faster biodegradation rates than samples with lower severity factor. This behaviour can be explained due to the decrease of ordered structures and sulphur bridge as a result of the SE process, which facilitates the biodegradation of feathers.

## 4. Conclusions

In this work, chicken feathers were successfully treated under different steam explosion conditions, obtaining samples with a brownish appearance and a lower process yield as the severity factor of the SE process increased. Obtained samples were characterized from the physicochemical and morphological point of view in order to investigate the influence of the SE conditions on feather structure and properties. Additionally, the biodegradation capacity of the treated feathers in soil was examined.

The results showed that the SE process led to treated feathers with higher apparent density compared to the untreated ones. Moreover, a modification of the secondary structures of the keratin was observed because of the SE treatment, obtaining a decrease of ordered β-keratin mainly being substituted by disordered domains. The elemental analysis revealed a decrease of the sulphur content with the increase of the severity, which can be correlated with the disruption of disulphide bonds and the formation of sulphur containing volatiles. Morphology wise, an increase of the rugosity of the feathers was observed as the severity of the SE increased, which can favour the adhesion of microorganisms and, thus, facilitate their biodegradation. From the X-ray diffraction, an increase of the relative crystallinity for low SE severities was observed, which abduce the removal of the amorphous phase. However, higher severity of the SE process led to a clear decrease of the relative crystallinity related to the destruction of the crystalline phase, owing to the more sever temperatures.

Finally, the biodegradation test revealed that feathers treated with SE showed higher and much faster biodegradation compared with the untreated ones. Regarding the influence of the SE severity, despite scarce differences that were observed as far as final biodegradation is concerned, the increase of the severity did lead to an increase of the biodegradation rate in the first days. These results open up the possibilities of using feather waste as a component for environmentally friendly agricultural bioplastics that can be degraded in-situ in soil.

## Figures and Tables

**Table 1:** Steam explosion conditions, particle size, yield, and bulk density of the SE process of treated feathers.

Samples	Steam Explosion			Grinding	Yield (%)	Bulk Density (g cm^−3^)
	Temperature (°C)	Residence Time (min)	Severity Factor	Particle Size (mm)		
Raw feathers-0.5 mm	-	-	-	0.5	-	0.015 ± 0.01
CF-SE-160 °C-2 min-0.5 mm	160	2	2.07	0.5	100	0.306 ± 0.02
CF-SE-160 °C-4 min-0.5 mm		4	2.37	0.5	80	0.447 ± 0.02
CF-SE-180 °C-2 min-0.5 mm	180	2	2.66	0.5	85	0.565 ± 0.04
CF-SE-190 °C-4 min-0.5 mm	190	4	3.25	0.5	80	0.706 ± 0.03

**Table 2:** Summary of the relative ratio of secondary structures in ATR-FTIR deconvolution of amide III band.

Samples	α-Helix (%)	β-Sheet (%)	β-Turns (%)	Random Coil (%)
Raw feathers-0.5 mm	13 ± 4	72 ± 3	10 ± 2	3 ± 1
CF-SE-160 °C-2 min-0.5 mm	18 ± 2	63 ± 4	3 ± 1	14 ± 4
CF-SE-160 °C-4 min-0.5 mm	20 ± 5	62 ± 2	5 ± 2	14 ± 5
CF-SE-180 °C-2 min-0.5 mm	28 ± 2	53 ± 5	3 ± 1	17 ± 4
CF-SE-190 °C-4 min-0.5 mm	29 ± 2	52 ± 1	4 ± 1	16 ± 3

**Table 3:** Relative crystallinity values determined for CF treated under different SE conditions.

Samples	Severity Factor	Relative Crystallinity (%)
Raw feathers-0.5mm	-	0.35 ± 0.01
CF-SE-160°C-2 min-0.5 mm	2.07	0.45 ± 0.01
CF-SE-160°C-4 min-0.5 mm	2.37	0.40 ± 0.02
CF-SE-180°C-2 min-0.5 mm	2.66	0.39 ± 0.01
CF-SE-190°C-4 min-0.5 mm	3.25	0.26 ± 0.02

## Data Availability

Data will be made available on request.

## Supplementary Materials

The following supporting information can be downloaded at: https://www.mdpi.com/article/10.3390/polym15183701/s1, Figure S1: Diagram of the Biodegradation test setup; Table S1: Elemental analysis of raw and treated feathers at different SE conditions; Table S2: Initial degradation and maximum degradation temperature of feather treated with different SE conditions; Figure S2: Representative deconvolution of XRD diffractograms of raw and SE feathers.
