# Supplementary material for: Enhanced Biodegradability in Soil of Chicken Feather by Steam Explosion for Potential Application in Agricultural Biodegradable Plastics
Source: Polymers (Basel). 2023 Sep 8;15(18):3701. doi: 10.3390/polym15183701 (PMC10537891; doi:10.3390/polym15183701)
Supplement: Supplementary file 1 [file polymers-15-03701-s001.zip › polymers-2532582-supplementary.pdf]

Supplementary material

Enhanced biodegradability in soil of chicken feather by steam explosion for potential application in agricultural biodegradable plastics

Julen Vadillo[1,*], Sarah Montes[1], Hans-Jürgen Grande[1,2], Steven Verstichel[3], Jonna Almqvist[4], Krystyna Wrześniewska-Tosik[5].

[1] *CIDETEC, Basque Research and Technology Alliance (BRTA), Paseo Miramón, 196,*

20014 Donostia-San Sebastián, Spain

[2] *University of the Basque Country (UPV/EHU), Advanced Polymers and Materials: Physics, Chemistry and Technology Department, Avda. Tolosa 72, 20018, Donostia-San Sebastian, Spain*

[3] *Normec OWS, Pantserschipstraat 1639000, Gent Belgium*

[4] *RISE Research Institutes of Sweden, Department of Biorefinery and Energy, S-892 50 Örnsköldsvik, Sweden*

[5] *Łukasiewicz Research Network- Łodz Institute of Technology, ul. Skłodowskiej-Curie 19/27 90-570 Łódź, Poland*

* Corresponding author: jvadillo@cidetec.es

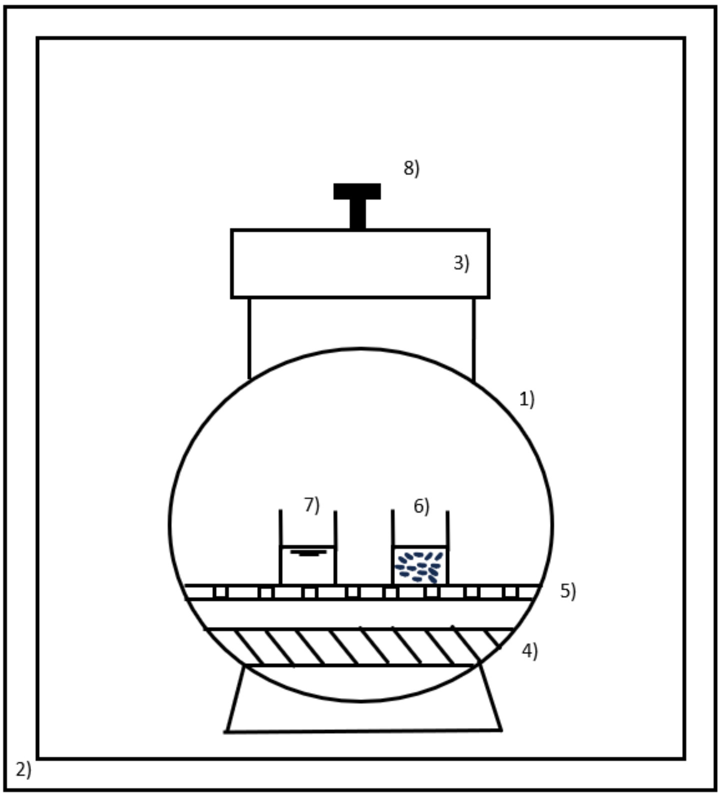

1) Static reactor
2) Incubation chamber
3) Airtight lid
4) Soil-test substance mixture
5) Perforated plate
6) Beaker with KOH
7) Beaker with water
8) Septum for gas analyses

Figure S1. Diagram of the Biodegradation test setup

Table S1. Elemental analysis of raw and treated feathers at different SE conditions.

Reference	Content (%)				
	N	C	H	S	Others
Raw chicken feathers	14.6	48.2	7.2	2.3	27.7
CF-SE-160°C-2min-0.5mm	14.4	48.2	7.3	2.2	27.9
CF-SE-160°C-4min-0.5mm	14.3	46.8	7.4	2.1	29.4
CF-SE-180°C-2min-0.5mm	14.4	47.6	7.3	2.0	28.7
CF-SE-190°C-4min-0.5mm	14.9	48.7	7.3	1.8	27.3

Table S2. Initial degradation and maximum degradation temperature of feather treated with different SE conditions

Reference	Severity factor	$Td_{5\%}$ (°C)	$Td_{10\%}$ (°C)	Td_{max1}(°C)
Raw Chicken feathers	-	53	165	316
CF-SE-160°C-2min-0.5mm	2.07	59	183	322
CF-SE-160°C-4min-0.5mm	2.37	63	185	321
CF-SE-180°C-2min-0.5mm	2.66	69	189	323
CF-SE-190°C-4min-0.5mm	3.25	74	190	324

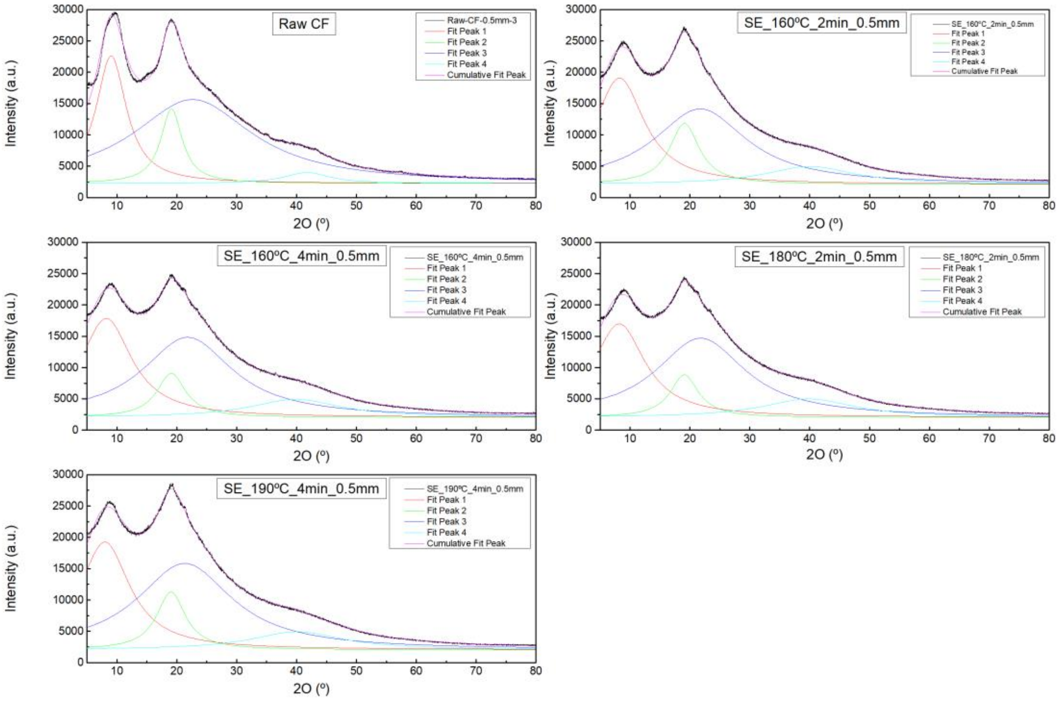

Figure S2. Representative deconvolution of XRD diffractograms of raw and SE feathers.
